# A novel microRNA signature for the detection of melanoma by liquid biopsy

**DOI:** 10.1186/s12967-022-03668-1

**Published:** 2022-10-15

**Authors:** Claudia Sabato, Teresa Maria Rosaria Noviello, Alessia Covre, Sandra Coral, Francesca Pia Caruso, Zein Mersini Besharat, Elena Splendiani, Laura Masuelli, Cecilia Battistelli, Alessandra Vacca, Giuseppina Catanzaro, Agnese Po, Andrea Anichini, Michele Maio, Michele Ceccarelli, Anna Maria Di Giacomo, Elisabetta Ferretti

**Affiliations:** 1 Department of Experimental Medicine, Sapienza University, 00161 Rome, Italy; 2 Biogem Scarl, Istituto di Ricerche Genetiche “Gaetano Salvatore”, 83031 Ariano Irpino, Italy; 3 Department of Electrical Engineering and Information Technology, University of Naples Federico II, Naples, Italy; 4 Center for Immuno-Oncology, Medical Oncology and Immunotherapy, Department of Oncology, University Hospital of Siena, 53100 Siena, Italy; 5 Medical Oncology, Department of Molecular and Developmental Medicine, University of Siena, 53100 Siena, Italy; 6 Epigen Therapeutics s.r.l., 53100 Siena, Italy; 7 Department of Molecular Medicine, Sapienza University, 00161 Rome, Italy; 8 Human Tumor Immunobiology Unit, Department of Research, Fondazione IRCCS Istituto Nazionale dei Tumori, ENETS Center of Excellence, Milan, Italy

**Keywords:** Melanoma, Liquid biopsy, microRNAs, Extracellular vesicles, Biomarkers signature, Diagnosis

## Abstract

**Background:**

Melanoma is the deadliest form of skin cancer and metastatic disease is associated with a significant survival rate drop. There is an urgent need for consistent tumor biomarkers to scale precision medicine and reduce cancer mortality. Here, we aimed to identify a melanoma-specific circulating microRNA signature and assess its value as a diagnostic tool.

**Methods:**

The study consisted of a discovery phase and two validation phases. Circulating plasma extracellular vesicles (pEV) associated microRNA profiles were obtained from a discovery cohort of metastatic melanoma patients and normal subjects as controls. A pEV-microRNA signature was obtained using a LASSO penalized logistic regression model. The pEV-microRNA signature was subsequently validated both in a publicly available dataset and in an independent internal cohort.

**Results:**

We identified and validated in three independent cohorts a panel of melanoma-specific circulating microRNAs that showed high accuracy in differentiating melanoma patients from healthy subjects with an area under the curve (AUC) of 1.00, 0.94 and 0.75 respectively. Investigation of the function of the pEV-microRNA signature evidenced their possible immune suppressive role in melanoma patients.

**Conclusions:**

We demonstrate that a blood test based on circulating microRNAs can non-invasively detect melanoma, offering a novel diagnostic tool for improving standard care. Moreover, we revealed an immune suppressive role for melanoma pEV-microRNAs.

**Supplementary Information:**

The online version contains supplementary material available at 10.1186/s12967-022-03668-1.

## Background

Melanoma is the most aggressive and deadly skin cancer. Though melanoma represents circa 1.8% of skin cancer, it accounts for over 80% of skin cancer deaths [1]. Recent studies describe an increasingly fast incidence of melanoma has been observed among developed countries [1]. World cancer statistics report a 5-year survival of 99.4% for stage I-II disease patients, followed by 68% for stage III patients and dropping to 29.8% for stage IV patients (https://seer.cancer.gov/statfacts/html/melan.html). Diagnosis of melanoma is performed through biopsy and pathological examination of the skin lesion, along with total skin and lymph node examination.

Even though, stage III and IV melanoma patients’ management has been transformed from an incurable disease with the use of targeted therapies and immune checkpoint inhibitors, still nearly 50% of unresectable or metastatic melanoma patients die in 5 years after treatment start [2–6].

Based on these data, metastatic melanoma necessitates efforts to identify new non-invasive biomarkers to improve diagnosis, staging, risk assessment and predict response to therapy [7]. In this context, microRNAs, a class of small non-coding RNAs involved in the epigenetic regulation of genes and key players in different cellular processes ranging from proliferation to cellular communication, are under evaluation as biomarkers. Indeed, many studies reveal that microRNAs-associated extracellular vesicles (EVs) are attractive candidates as disease biomarkers since they demonstrate dynamic changes related to disease status [8–10]. EV-microRNAs present several features that make them ideal candidates as biomarkers, namely their stability in biological fluids, easy detectability and rapid assessment.

Melanoma cells, as most malignant cancer cells, secrete EVs into the bloodstream [11, 12] and these EVs contain microRNAs [13].

Researchers have employed blood-based liquid biopsy in metastatic melanoma patients which allowed the detection of circulating microRNAs from different types of samples, including serum, plasma [14, 15] and plasma EVs (pEVs) [12, 16–22].

Of note, studies on pEV-microRNAs from melanoma samples are limited to date and the ones that have been performed report preliminary data from single patients cohorts without reaching a general consensus on the use of specific pEV-microRNAs as biomarkers [12, 16–22].

Therefore, in order to address the current medical need for non-invasive biomarkers in melanoma, pEV-microRNA expression levels were evaluated and a melanoma-specific pEV-microRNA biomarker signature was determined along with its diagnostic value.

## Methods

### Patients and samples

The study was carried out in three phases: one discovery phase and two validation phases.

The discovery cohort comprised samples from 19 patients with unresectable Stage III or IV cutaneous melanoma and measurable lesions by computed tomography (CT) or magnetic resonance imaging (MRI) scans. Patients were recruited in the phase Ib NIBIT-M4 study at the Center for Immuno-Oncology of Siena [23] and were treated with ipilimumab and guadecitabine (ClinicalTrials.gov Identifier NCT02608437). Plasma samples from 16 out of the 19 patients, collected before receiving treatments, were used for circulating microRNAs profiling analysis and this cohort was defined as the discovery cohort.

Twenty-two normal subjects, matched for age and gender, with no history of malignant disease, were recruited as controls (Ctrl). Characteristics of study participants of the discovery cohort are reported in Table 1.

**Table 1:** Characteristics of melanoma patients and normal subject controls of the discovery cohort

	Melanoma patients	Normal controls
	(N=16)	(N=22)
Gender		
Male	14 (12,5%)^a^	10 (45,5%)^a^
Female	2 (87,5%) ^a^	12 (54,5%) ^a^
Age (range)		
Male	58 (27-82) ^b^	44 (21-67) ^b^
Female	54 (50-59) ^b^	48,5 (22-75) ^b^
BRAF status		
Mutated	5 (31,3%) ^a^	–
Wild-type	11 (68,7%) ^a^	
M stage		
M0	2 (12,5%) ^a^	
M1a	8 (50%) ^a^	–
M1b	1 (6,3%) ^a^	
M1c	5 (31,2%) ^a^	
LDH		
≤ULN	14 (87,5%) ^a^	–
>ULN	2 (12,5%) ^a^	
Prior lines of therapy		
0	16 (100%) ^a^	–
1	0	

Number of prior lines of therapy: 0 indicated no prior lines of therapy, 1 indicated prior lines of therapy. Enrolled patients did not receive prior lines of therapy

*LDH* lactate dehydrogenase, *UNL* upper limit normal

^a^ *n* (%), ^b^ Median (range).

A second independent internal cohort of 20 Stage IV melanoma patients, enrolled within the NIBIT-M2 study [24] (ClinicalTrials.gov Identifier NCT02460068), was used as the internal validation cohort. Plasma samples were collected before patients received any treatment. Eighteen age- and gender-matched normal subjects were used as Ctrl of the validation cohort (Table 2).

**Table 2:** Characteristics of melanoma patients and normal subject controls of the independent internal validation cohort

	Melanoma patients (N=20)	Normal control (N=18)
Gender		
Male	13(65%)^a^	11 (61,1%)^a^
Female	7 (35%)^a^	7 (38,9%)^a^
Age (range)		
Male	58(43-79)^b^	58 (41-67)^b^
Female	48 (20-67)^b^	53 (21-65)^b^
BRAF status		
Mutated	8 (40%)^a^	–
Wild-type	8 (40%)^a^	
Unknown	4 (20%)^a^	
M stage		
M1c	20 (100%)^a^	
Number of brain lesions		
1	7 (35%)^a^	–
2	5 (25%)^a^	
3	5 (25%)^a^	
>3	3 (15%)^a^	
Previous local treatments for brain metastases		
Surgery	5 (25%)^a^	–
Radiotherapy	2 (10%)^a^	
LDH		–
≤ULN	15 (75%)^a^	
>ULN	5 (25%)^a^	

*LDH* lactate dehydrogenase, *UNL* upper limit normal

^a^ *n* (%)

^b^ Median (range).

Written informed consent was obtained from all participants and the study was approved by the institutional review board of each participating institution.

The publicly available dataset (GSE20994), retrieved from the Gene Expression Omnibus (GEO) database, included microRNAs expression levels from 35 peripheral whole blood samples collected using PAXgene Blood RNA tubes (BD, Franklin Lakes, New Jersey USA) from melanoma patients [0-I clinical stages: 23 patients (65.7%); II clinical stage: 8 patients (22.9%); III-IV clinical stages: 4 patients (11.4%)] and from 20 healthy subjects.

### pEV isolation

Plasma samples were collected in EDTA-treated tubes and processed within 30 minutes. To eliminate the risk of bias related to hemolysis, samples were visually assessed and hemolyzed, icteric, or lipemic samples were excluded. Hemolysis was further assessed using miR-23a/miR-451 ratio, as described in the RNA isolation section. Three out of 19 patient samples of the discovery cohort did not satisfy the criteria and were excluded from the analysis. Two-hundred fifty microliters (µl) of plasma were precleared with thrombin (Cat#TMEXO-1, System Biosciences SBI, Palo Alto, CA) and then used for EV precipitation by ExoQuick Ultra EV Isolation Kit for Serum and Plasma according to the manufacturer’s protocol (Cat # EQULTRA-20A-1 System Biosciences SBI, Palo Alto, CA). The quality of isolated pEV was assessed using transmission electron microscopy (TEM) and the number of particles was quantitated by Tunable Resistive Pulse Sensing (TRPS) measurement. Common microvesicle markers were detected by western blot.

### pEV characterization

Transmission electron microscopy (TEM) of pEVs was performed as previously described [25]. Briefly, pEVs were fixed in 4% paraformaldehyde and adsorbed on formvar-carbon-coated copper grids. The grids were then incubated in 1% glutaraldehyde for 5 minutes, washed with deionized water eight times, and then negatively stained with 2% uranyl oxalate (pH 7) for 5 minutes and methyl cellulose/uranyl for 10 minutes at 4°C. Excess methyl cellulose/uranyl was blotted off, and the grids were air-dried and observed in TEM (Morgagni 268D, Philips Electronics, Eindhoven, The Netherlands) at an accelerating voltage of 80 kV. Digital images were taken with Mega View imaging software.

The particle size distribution and concentration of purified EVs from plasma samples of healthy donor and melanoma patient were determined by Exoid Tunable Resistive Pulse Sensing (TRPS) measurement system (Izon Science, USA) and analyzed with Izon Control Suite Software (version 1.0.2.32). To determine the particle concentration, for each sample, an average of 500 particles were counted and compared with the size and concentration reference calibration particles (Izon Science, USA). A 150 nm polyurethane nanopore membrane (Izon Science, USA) was stretched at 47 mm by applying the pressure at 300 Pa and voltage at 700 mV.

For western blot analysis, resuspended pEVs were lysed in an appropriate volume of 1X RIPA buffer (50 mM Tris-HCl pH 7.6, 0.5% Sodium deoxycholate, 1% NP40, 0.1% SDS, 140 mM NaCl, 5 mM EDTA pH 8, 100 mM NaF, 2 mM Na_4_P_2_O_7_) plus complete protease inhibitor mixture (Cat# S8820, Sigma) on ice for 30 minutes followed by centrifugation at 13000 RPM for 30 minutes. pEV lysates were resolved by SDS–PAGE and transferred to nitrocellulose membranes (NBA085C001EA, PerkinElmer, Waltham, MA, USA). After membrane blocking with 5% non-fat dry milk in Tris-buffered saline with 0.1% Tween-20 detergent (TBS-T), membranes were incubated overnight with the following specific antibodies: CD63 (VPA00798; BioRad), CD81 (sc-166029; Santa Cruz Biotechnology), Calnexin (sc-46669; Santa Cruz Biotechnology), HSP70 (sc-33575; Santa Cruz Biotechnology) and TSG-101 (HPA-006161; Atlas Antibodies). A horseradish peroxidase (HRP)-conjugated secondary antibody (Bethyl Laboratories, Inc.) and an enhanced chemiluminescence kit (K-12045-D50; Advasta Inc. San Jose, CA, USA) were used to reveal immunoreactivity. Images were acquired with Azure Biosystem C600 (Azure Biosystems, Inc., Dublin, CA, USA).

### RNA isolation

pEVs were processed for RNA isolation using the “Maxwell RSC miRNA plasma and serum kit” (Cat# AS1680; Promega). Three synthetic spike-ins (Ath-miR-159a, Cel-miR-254, osa-miR-414) were added to samples after lysis to assess RNA isolation efficiency. Isolated RNA was subjected to quality control for hemolysis detection using the ratio of miR-23a to miR-451 [26–28]. Two microliters of RNA were reverse transcribed to complementary DNA (cDNA) using the TaqMan Advanced MicroRNA cDNA synthesis Kit (Cat# A28007; ThermoFisher Scientific, Rockford, USA) according to the manufacturer's protocol.

### pEV-microRNAs profiling analysis

cDNA samples were profiled using the RT-qPCR panel of 754 microRNA TaqMan Advanced miRNA Human A and B Cards, according to manufacturer instructions (Cat# A31805; ThermoFisher Scientific, Rockford, USA). Real-time PCR reactions were carried out on the ViiA 7 Real-Time PCR System (384-well configuration, Applied Biosystem) using the following thermal protocol: 10 minutes enzyme activation at 92 ˚C followed by 40 cycles of 1 second denaturation at 95 ˚C and 20 seconds annealing/elongation at 60 ˚C.

Analysis of pEV-microRNAs expression levels was performed using the R environment (http://www.r-project.org/). Data were cleaned, filtered, and normalized. Expression analysis was performed using the Bioconductor package HTqPCR [29]. Specifically, the RT-PCR cycle thresholds, Ct, that were “Undetermined” were assigned a value of “Ct=40”. pEV-microRNAs with Ct values > 33 were considered not expressed. pEV-microRNAs with Ct values ≤ 33 were considered informative and included in subsequent data expression analysis. To find the best normalization strategy, five normalization methods (quantile, scale.rank invariant, norm.Rank invariant, geometric mean, deltaCt) were tested. The norm.Rank invariant normalization method provided a lower coefficient of variation (CV) and standard deviation (SD), across all samples, compared to the other methods and was selected as the most appropriate method. Differential expression analysis of pEV-microRNAs between Patients and Ctrl was conducted on normalized data, calculating the delta-delta Ct difference and reporting the fold change. Those pEV-microRNAs with fold change >|2| and p < 0.05 were considered as differentially expressed between Patients and Ctrl. Differentially expressed pEV-microRNAs were used as input data for hierarchical clustering.

### Gene Ontology Enrichment analysis

Gene Ontology (GO) enrichment analysis of differentially expressed pEV-microRNAs was computed using the clusterProfiler R package [30]. GO enrichment results were visualized using an R custom script. Enriched GO terms with a false discovery rate (FDR) and adjusted p-value cutoff of 0.01 were identified and used for biological interpretation. In order to simplify the enriched results, the similarity of GO terms was computed and those highly similar (similarity cutoff of 0.7) were hierarchically clustered keeping the term with the highest score as the representative of each group.

### Identification of pEV-microRNA signature in metastatic melanoma patients

Penalized logistic regression was performed on microRNAs data of the discovery cohort to determine the best pEV-microRNA predictors of melanoma status using the gmlnet R package [31]. The model was built on Ct values using differentially expressed pEV-microRNAs (Patients vs. Ctrl, Fold change Ct = 2). The least absolute shrinkage and selection operator (LASSO) regularization was applied to find a pEV-microRNA signature minimizing the number of features. Ten-fold cross-validation was applied to select the optimal shrinkage parameter, which gives the most regularized model such that error is within one standard error of the minimum [31].

A bootstrap approach was used by resampling with replacement 10000 times the discovery cohort and then applied the LASSO model for assess the reliability of the generated signature. A p-value was computed by estimating the frequency of occurrence of at least the 50% of the original signature in all the signatures generated from the bootstrap resampling.

Logistic regression with the resulting signature was used to classify patients with metastatic melanoma. The classification performance was measured using leave-one-out cross-validation (LOOCV). The Receiver operating characteristics (ROC) and Precision-Recall (PR) curves were generated with the pROC R package [32]. The optimal probability threshold was determined by minimizing the false positive rate in the classification. Sensitivity, specificity, overall classification accuracy and area under the ROC and PR curves were computed to assess classification performances.

### Validation of pEV-microRNA signature in the public external dataset

The logistic regression model with the pEV-microRNA signature was validated in the public external independent test dataset (GSE20994), retrieved from the Gene Expression Omnibus (GEO) database. An optimal probability threshold specific for this dataset was determined again by minimizing the false positive rate in the classification, due to the mixed tumor stage nature of the cohort

and the overall performance on the test set was then evaluated with the same previous classification metrics. A ROC curve was generated from the prediction analysis in R.

To further test if the four pEV-microRNA signature could be identified by chance, the performance accuracies of 1000 random models with random microRNA signatures were computed. The resulting accuracy distribution was compared with the pEV-microRNA signature model accuracy and a p-value was computed.

### pEV-microRNA signature in internal validation cohort by droplet digital PCR

Four pEV-microRNAs were validated in the independent internal validation cohort, as described above (Table 2). pEV-microRNAs levels were quantified using droplet digital PCR (ddPCR) (Bio-Rad Laboratories, Hercules, CA). For each sample, microRNAs were individually reverse-transcribed starting from 5 µl of RNA using TaqMan™ MicroRNA Reverse Transcription Kit (Cat# 4366596, Applied Biosystem) and TaqMan™ MicroRNA Assays (Cat# 4427975, ThermoFisher Scientific) following manufacturer's protocol (ID: 001023 hsa-miR-412-3p; ID: 001051 hsa-miR-507; ID: 002877 hsa-miR-1203; ID: 002117 hsa-miR-362-3p; ThermoFisher Scientific). Then, 8 μl of each diluted cDNA template were added to a ddPCR reaction mixture containing 11 μl of ddPCR Supermix for Probes (no dUTP) (Cat# 1863025; Bio-Rad Laboratories), 1.1 μl of TaqMan specific miRNA probe (Cat# 4427975, ThermoFisher Scientific) and 1.9 μl nuclease-free water. For droplets generation, 20 μl of each ddPCR reaction mixture was loaded in disposable droplet generator cartridges (Cat# 1864008; Bio-Rad Laboratories) along with 70 μl of droplet generation oil for probes (Cat#1863005; Bio-Rad Laboratories) and placed into QX200^TM^ Droplet Generator (Bio-Rad Laboratories). Generated droplets were transferred to 96-well PCR plates (Cat# 12001925; Bio-Rad Laboratories). PCR was carried out in a C1000 Touch Thermal Cycler (Bio-Rad Laboratories) using the following thermal cycling program: 10 minutes at 95 °C for enzyme activation, followed by 45 cycles of 30 seconds at 94 °C and 1 minute annealing/extension step at the appropriate temperature based on the primer/probe set (in our case at 58 °C for miR-362-3p and 56°C for the remaining 3 microRNA), 10 minutes at 98 °C for enzyme deactivation followed by infinite hold at 4 °C. Finally, the QX200 Droplet Reader (Bio-Rad Laboratories) was used to read the fluorescence signals, and QuantaSoft software was used for ddPCR data analysis (version 1.7.4; Bio-Rad Laboratories).

### Cell-type enrichment analysis of pEV-microRNA signature

In order to evaluate the cellular origin of the four pEV-microRNAs, the count per million (CPM) expression profiles in several human cell lines and tissues from the FANTOM5 database were retrieved [33]. The heatmap of z-scores of log10 average expression values of the pEV-microRNA signature in these cellular compartments and tissues has been generated (no data available in FANTOM5 database for miR-1203).

### Experimental validation of microRNA target gene

HEK293T cells were plated in 24-well plates in Dulbecco’s Modified Eagle’s

Medium-high glucose (D6546, Sigma Aldrich), supplemented with 10% fetal bovine serum (FBS, Sigma Aldrich), 2 mM L-glutamine (Sigma Aldrich) and 100 units·mL^−1^ antibiotic solution (100 units·mL^−1^ penicillin and 10000 µg·mL^−1^ streptomycin, Sigma Aldrich). Cells were cultured at 37°C in a humidified 5% CO_2_ atmosphere.

MicroRNA targets prediction was identified by bioinformatics analysis using the online miRNA Pathway Dictionary Database (miRPathDB) at https://mpd.bioinf.uni-sb.de/.

Human 3’ UTR of TNF Superfamily Member 4 (TNFSF4) cloned downstream of the secreted firefly luciferase reporter gene was obtained from GeneCopoeia (Cat# HmiT110979-MT06). 100 ng of 3’ UTR of TNFSF4 were transiently co-transfected into HEK293T cells with 50 nM of the following miRIDIAN microRNA mimic (Dharmacon): mimic negative controls (Cat# CN-001000-01-05), hsa-miR-412-3p (Cat# C-300739-03-0005), hsa-miR-507 (Cat# C-300847-05-0005), hsa-miR-1203 (Cat# C-301329-00-0005) by using Lipofectamine™ 2000 (Invitrogen, Thermo Scientific). Twenty-four hours after transfection, cells were harvested and assessed for the Firefly Luciferase Assay 2.0 (Cat# 30085, Biotium). Cells were incubated for 15 min at room temperature on orbital shaker with 100 µL of 1× Passive Lysis Buffer. Then, 20 µL of the cell lysate was tested with 100 µL of Firefly and Renilla solution in a 96-well plate. Luciferase activity was detected with a luminometer (Promega GloMax Plate Reader). Results were expressed as the ratio of Firefly luciferase to Renilla activity. Reported values are means ± S.D. of values from three experiments, each performed in triplicate.

### Statistical analysis

Statistical analyses on differentially expressed pEV-microRNAs were conducted on R programming using the Wilcoxon Mann-Whitney test and considered statistically significant when p values were below <0.05. Statistical analyses for ddPCR and luciferase reporter assay were performed using GraphPad Prism software Version 8.3 (La Jolla, California, USA). Wilcoxon Mann-Whitney statistical test was used to compare pEV-microRNA levels between experimental groups. Student’s unpaired t-test was used to determine significant differences between the effect of each miRNA on the TNFSF4 3'UTR. P values < 0.05 were considered statistically significant.

## Results

### pEV-isolation and pEV-microRNAs profiles in the discovery cohort

Plasma samples collected from the discovery cohort were from metastatic melanoma patients and healthy controls (Ctrl), matched for age and gender, as reported in Table 1.

**Fig. 1:**
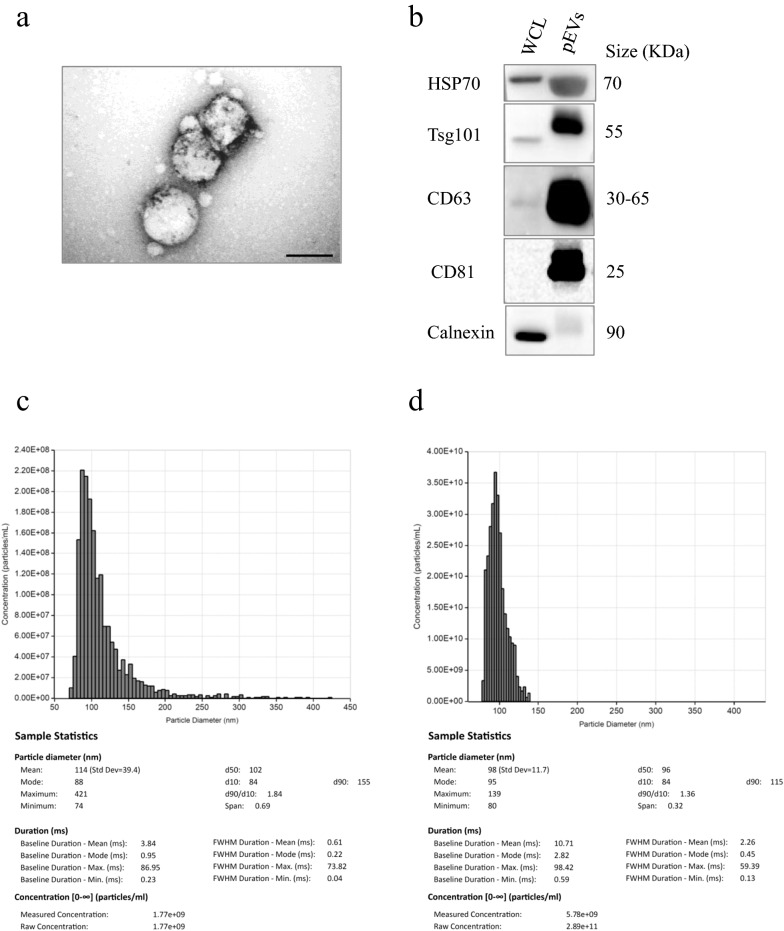
Characterization of pEVs in melanoma patients (Patient) and normal subjects (Ctrl). **A** Transmission electron microscopy visualization of EV isolated from human plasma samples. Isolated EV displayed multiple vesicles with a round-shaped morphology and a diameter of 100-400 nm. Scale bars correspond to 200 nm. **B** Western blot analysis of common exosomal markers (HSP70, TSG101, CD63 and CD81) and cell organelle (calnexin) in whole cell lysate (WCL) of WM793 melanoma cells and EV isolated from normal subject control (Ctrl) plasma sample. WCL was loaded as positive control. **C**, **D** Size distribution and concentration of isolated pEVs from healthy donor (**C**) and melanoma patient (**D**) using Tunable Resistive Pulse Sensing method

Plasma samples were used for EV isolation and characterized by TEM, western blot and Exoid Tunable Resistive Pulse Sensing (TRPS) method. Ultrastructural analysis of pEVs indicated the presence of membrane rounded-shaped vesicles with a diameter between 100 and 400 nm (Figure 1A). The presence of pEVs in the preparation was also confirmed by the positive staining for the canonical microvesicle markers (HSP70, TSG101, CD63 and CD81) and absence of the intracellular marker calnexin (Figure 1B).

The TRPS measurements of the pEVs isolated from healthy donor revealed a particles concentration of 1.77e+09 particles/ml and a particle diameter of 114 nm (standard deviation of 39.4), as reported in Figure 1C. A higher particles concentration (2.89e+11 particles/ml) was detected for pEVs isolated from melanoma patient, owning a particle diameter of 98 nm (standard deviation of 11.7) (Figure 1D).

Based on these findings, the successful pEV isolation method allowed us to proceed with RNA isolation to then analyze microRNA expression levels.

The pEV-microRNA expression profile was performed using a panel of 754 microRNAs. After data filtering, a total of 545 pEV-microRNAs were detected in all samples (Additional file 1: Figure S1). Principal component analysis (PCA) of raw (Additional file 1: Figure S2A) and normalized (Additional file 1: Figure S2B) pEV-microRNA expression data allowed us to clearly distinguish Ctrl and melanoma patients.

**Fig. 2:**
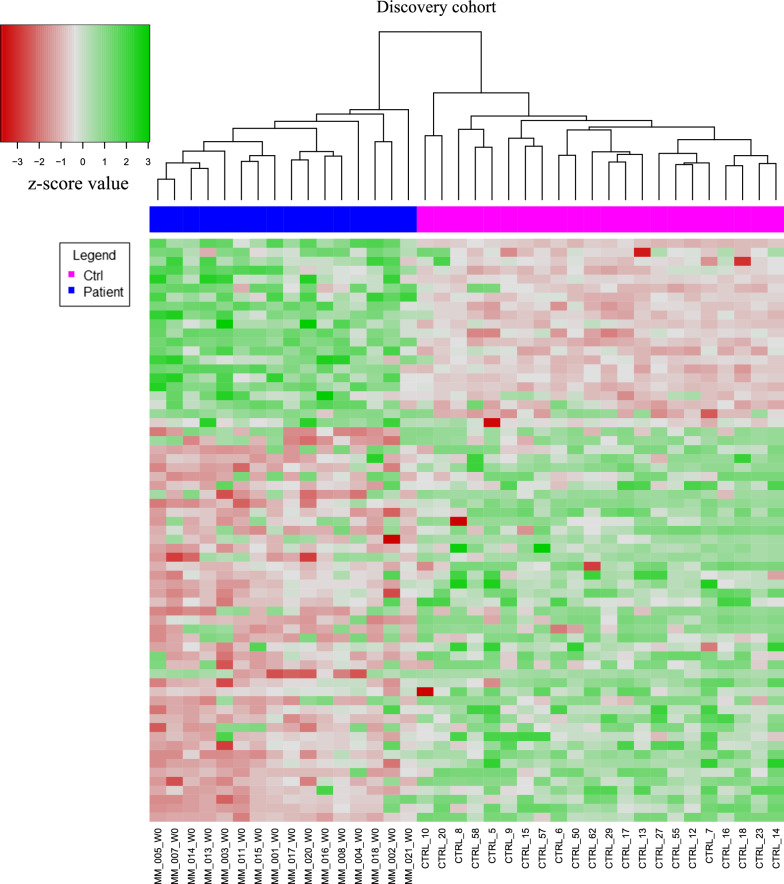
pEV-microRNA profiles in melanoma patients (Patient) and normal subjects (Ctrl). Heatmap of 65 differentially expressed pEV-microRNAs (21 down-regulated in Patients vs Ctrl, 44 up-regulated in Patients vs Ctrl), in melanoma patients (blue) and normal subject control (purple) with a statistically significance of p<0.05. Each row represents an individual microRNA, each column represents an individual sample

We performed differential expression analysis between patients and Ctrl, obtaining 65 differentially expressed (DE) pEV-microRNAs (Additional file 2: Table S1). Specifically, 44 pEV-microRNAs resulted up-regulated and 21 pEV-microRNAs were down-regulated in patients versus Ctrl, as shown in the heatmap of Figure 2, illustrating a clear separation of melanoma patients and Ctrl in two expression clusters.

To investigate the biological processes in which the 65 DE pEV-microRNAs were involved, Gene Ontology (GO) enrichment analysis was further performed. Specifically, up-regulated and down-regulated pEV-microRNAs were analyzed separately, the results of GO Biological Processes (GO-BP) enrichment analysis are shown in Additional file 1: Figures S3 and S4. Up-regulated pEV-microRNAs were involved in the regulation of endothelial cell migration and proliferation, regulation of cell motility, angiogenesis and regulation of necrotic cell death (Additional file 1: Figure S3). Whereas the down-regulated pEV-microRNAs were characterized by functions related to the regulation of oncogenic signaling pathways, as TGF-β and NFkB pathways, regulation of mitotic cell cycle and cell differentiation, in addition to the regulation of vasculature development (Additional file 1: Figure S4).

These results allowed the definition of a pEV-microRNA profile that discriminates metastatic melanoma patients from normal subjects.

### pEV-microRNA diagnostic signature

**Fig. 3:**
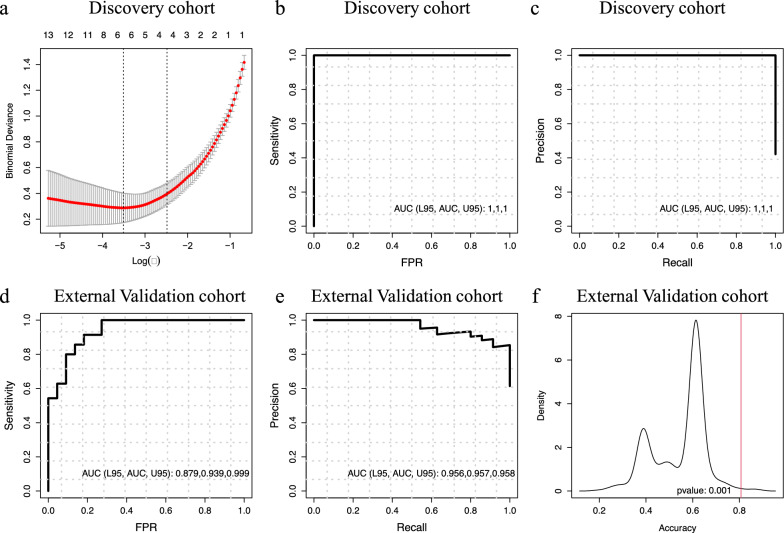
Evaluation of four microRNA signature predictive performance. **A** Parameter selection in LASSO regression. **B**, **C** ROC and PR curves for discovery cohort. **D**, **E** ROC and PR curves for external validation (GSE20994) cohort. **F** Comparison of pEV-microRNA signature model accuracy with an accuracy distribution of 1000 models with random microRNA signatures

With the aim to identify pEV-microRNA melanoma-specific signature with diagnostic value, the least absolute shrinkage and selection operator (LASSO) logistic regression analysis was performed by using the 65 DE pEV-microRNAs as input (Figure 3A, Additional file 1: Figure S5). The optimal minimum tuning parameters *λ* that gives minimum mean cross-validated error of 0.03 with log(λ) = −3.5 (Figure 3A) and four non-zero coefficients were chosen: miR-1203*(-1.24) + miR-412-3p*(-0.55) + miR-507*(-0.21) + miR-362-3p *(4.03).

To assess the uncertainty associated with the signature generation, we estimated the frequency of occurrence of at least the 50% of the identified signature in all the signatures generated from a bootstrap resampling approach. The resulting significant p-value of 0.04 statistically supported the LASSO signature estimator.

The model with the selected 4 pEV-microRNAs was able to discriminate metastatic melanoma patients with respect to Ctrl, namely miR-412-3p, miR-507, miR-1203 and miR-362-3p (Figures 3B and C). Specifically, miR-412-3p, miR-507 and miR-1203 were expressed at higher levels in metastatic melanoma pEVs, while miR-362-3p was expressed at lower levels in metastatic melanoma pEVs (Additional file 2: Table S1).

To evaluate the cellular origin of the identified pEV-microRNAs signature, we interrogated the FANTOM5 miRNA expression atlas where mature microRNAs abundances have been profiled across primary human cells and tissues [33]. Interestingly, we found that miR−412−3p is expressed in several cell lines and tissues, while miR-507 and miR-362-3p showed an expression enrichment in melanocytes (Additional file 1: Figure S6). No data was available in FANTOM5 database for miR-1203.

As reported in Table 3, the identified pEV-microRNA signature demonstrated high diagnostic performance in the discovery cohort.

**Table 3:** Predictive performance evaluation of pEV-microRNA signature in discovery and validation cohorts

Metrics	Discovery cohort	External validation cohort (GSE20994)
Accuracy	1.00	0.81
Sensitivity	1.00	1.00
Specificity	1.00	0.50
Positive Prediction Rate	1.00	0.76
Negative Prediction Rate	1.00	1.00
AUC ROC (95% CI)	1.00 (1.00, 1.00)	0.94 (0.88, 0.99)
AUC PR (95% CI)	1.00 (1.00, 1.00)	0.96 (0.96, 0.96)

These results allowed us to identify a melanoma-specific pEV-microRNA signature, able to differentiate metastatic melanoma patients from normal subjects.

### pEV-microRNA signature validation in an independent cohort from public dataset

In order to assess the accuracy of the 4 pEV-microRNA signature as a diagnostic tool, we analyzed an independent external dataset of circulating microRNAs (Gene Expression Omnibus (GEO) database; GSE20994) from normal controls (n = 20) and mixed melanoma patients (n = 35, 0-I clinical stages: 23 patients (65.7%); II clinical stage: 8 patients (22.9%); III-IV clinical stages: 4 patients (11.4%).

Due to the different nature in terms of tumor stage composition of the external dataset regarding to our discovery cohort, we used an ad hoc threshold of prediction that was specific for the validation cohort. The performance of the 4 pEV-microRNA signature to discriminate the patients from controls resulted to be high with an accuracy of 0.81 (Table 3) (vs. an accuracy of 0.71 by using the discovery cohort threshold) and AUCs of 0.94 (95% CI: 0.88, 0.99) and 0.96 (95% CI: 0.96, 0.96) for ROC and PR curves, respectively (Figures 3D and E, Table 3). The results implied that the performance of our model was robust and accurate also in a more clinically heterogenous cohort. To further test if the four pEV-microRNA signature could be identified by chance, the performance accuracies of 1000 models with random microRNA signatures were computed and compared with the pEV-microRNA signature model accuracy. The resulting highly significant p-value of 0.001 statistically supported the pEV-microRNA signature identification (Figure 3F).

In conclusion, the ability of melanoma-specific pEV-microRNA signature to identify melanoma patients was robustly validated in an independent cohort of heterogeneous melanoma patients.

### pEV-microRNA signature validation in an independent internal cohort

**Fig. 4:**
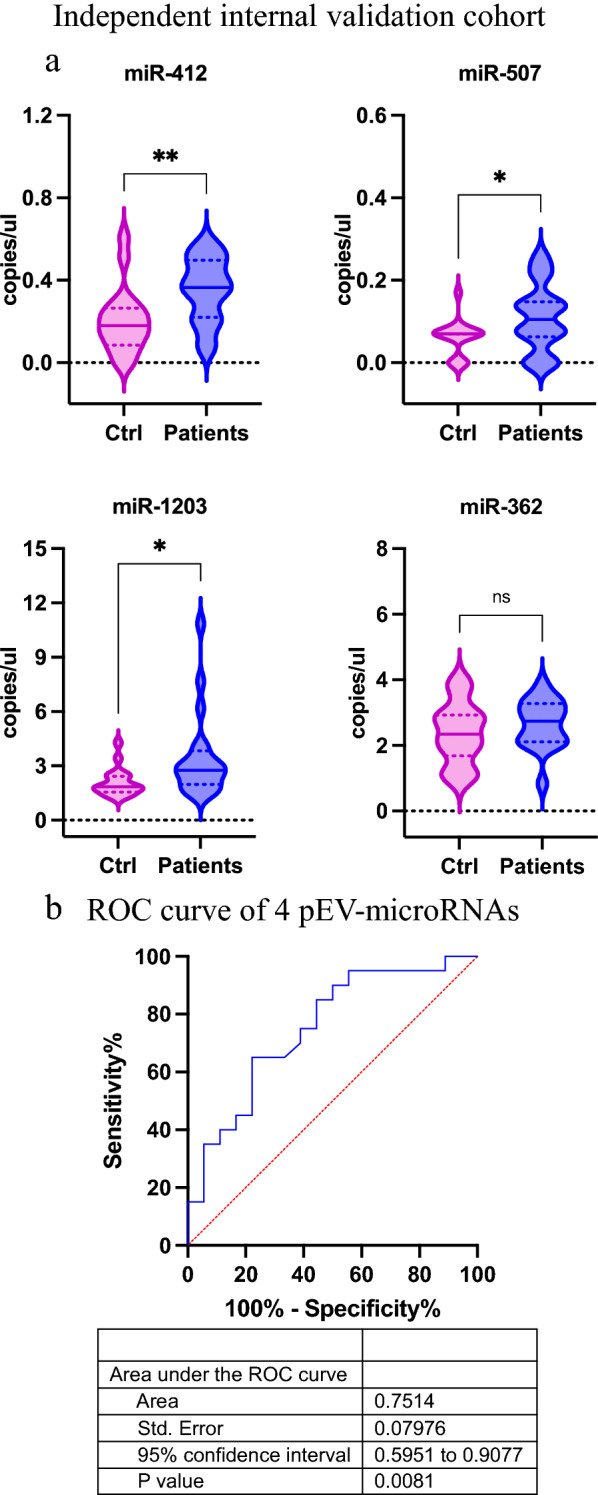
Absolute quantification of 4 pEV-microRNA and evaluation of diagnostic performance in an independent internal cohort. **A** Absolute quantification of circulating levels of 4 pEV-microRNAs (miR-412-3p; miR-507; miR-1203 and miR-362-3p) in an independent internal cohort of normal control (Ctrl) and metastatic melanoma patients (Patients) using ddPCR. Significant differences are highlighted with a starlike symbol (* p value <0.05, **, p value < 0.005), whereas not statistically significant difference with the abbreviation of “ns”. **B** ROC curves and AUC of 4 pEV -microRNAs in an independent internal cohort (AUC=0.75, p-value=0.008)

To further validate the ability of the 4 pEV-microRNA signature to differentiate melanoma patients from normal controls, we quantified their expression levels in an independent internal cohort of metastatic melanoma patients (n = 20) and controls (n = 18), matched for age and gender, as reported in Table 2, and evaluated their diagnostic power. The ddPCR, that allows an absolute quantification, was used for the evaluation of circulating pEV-microRNAs in this cohort. As reported in Figure 4A, miR-412-3p, miR-507 and miR-1203 resulted significantly deregulated, while miR-362-3p was not statistically significant. Nevertheless, the diagnostic performance of the 4 pEV-microRNAs was evaluated and resulted in an AUC of 0.75 (Figure 4B). The difference in the diagnostic performance among the two validation cohorts can be explained both by the different cohorts that were used as well as the different detection technologies that were employed. Results from this set of experiments allowed us to validate the signature for the identification of metastatic melanoma patients.

### Immunoregulatory role of circulating miR−412−3p, miR-507 and miR-1203

**Fig. 5:**
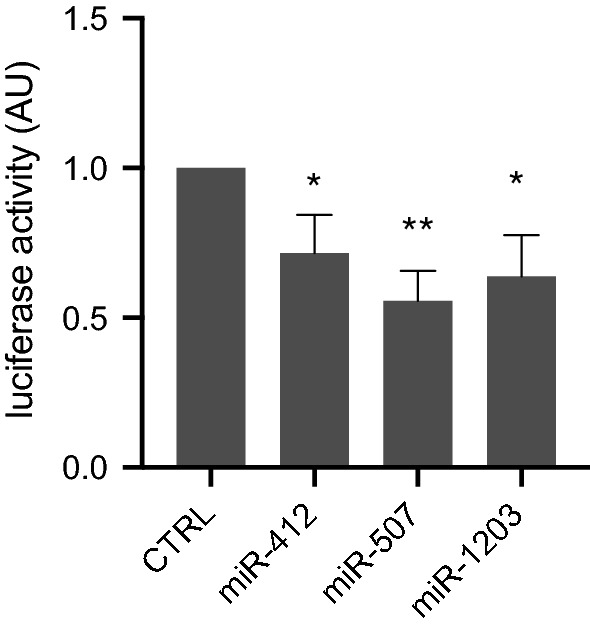
MicroRNA target genes experimental validation. Relative Luciferase activity in the HEK293T cell line following co-transfection of the 3’UTR of TNFSF4 with miR-412-3p, miR-507, and miR-1203. Values represent the means ± S.D. of values from three experiments, each performed in triplicate

A previous publication reported that EVs from melanoma patients are characterized by low levels of immunoregulatory proteins respect to EV from healthy subjects [22]. Therefore, we interrogated online databases searching for microRNA targets (miRNA Pathway Dictionary Database, miRPathDB), focusing on those genes coding for the immunoregulatory proteins that are down-regulated in EV from melanoma patients. Indeed, we found that the gene TNFSF4, coding for the immunoregulatory protein OX40L [22], was a putative target of 3 of the pEV-microRNA melanoma signature, namely miR-412-3p, miR-507 and miR-1203. We experimentally validated the binding of the pEV-microRNAs by luciferase reporter assays. Indeed, results showed that all 3 microRNAs bind the 3’UTR of TNFSF4, with a statistically significant reduction of luciferase activity for miR-412-3p, miR-507 and miR-1203, as reported in Figure 5. These evidences support the hypothesis that the high level of these circulating microRNAs in melanoma patients can dampen melanoma immunogenicity and suggest an immune suppressive role of these pEV-microRNAs.

## Discussion

Improvement of melanoma patients’ management is an urgent medical need. In this scenario blood-based liquid biopsy has received a lot of attention due to its non-invasive nature and power [34]. Indeed, circulating biomarkers allow continuous disease monitoring and patient management, having a remarkable clinical application especially for advanced cancers.

Circulating biomarkers are enriched in EVs, cell-derived membranous structures released by cells [35], whose cargo content includes microRNAs and whose dynamic changes have been related to different disease status [8–10].

MicroRNAs, a class of small non-coding RNAs, are particularly stable in biological fluids, their dysregulation in cancer has been widely reported and specific microRNA pattern expressions reflect cancer tissue [36–39]. Therefore, pEV-microRNAs are a powerful source for discovering new disease biomarkers. Of note, few reports have been published regarding the specific scenario of pEV-microRNAs in melanoma with no conclusive indication on their diagnostic role [12, 16–19] and for this reason we focused our study on this aspect.

In the present study, we used a high-throughput PCR-based method to profile pEV-microRNAs in the discovery cohort of metastatic melanoma patients. Sixty-five pEV-microRNAs characterized melanoma patients with active disease with respect to controls. Pathway enrichment analysis of those microRNAs showed their involvement in regulating endothelial cell proliferation, migration and motility, angiogenesis and oncogenic signaling pathways, as TGF-β and NF-kappa B [40–42]. We next applied a microRNA bioinformatic feature selection to identify the melanoma-specific pEV-microRNA signature identifying a 4 pEV-microRNA panel, namely miR-412-3p, miR-507, miR-1203 and miR-362-3p with a high diagnostic power.

Furthermore, the 4 pEV-microRNA signature was successfully validated using two different methodological approaches in two independent cohorts with a good diagnostic performance.

Interestingly, the diagnostic performance of these microRNAs was high in cohorts that included different tumoral stages, underlying its power and reliability in an extended spectrum of melanoma patients.

Recently, a research group demonstrated that pEV from melanoma patients are secreted in part by residual or relapsing tumor cells, highlighting the use of pEV markers as predictive biomarkers [43]. Therefore, based on the specific medical needs for melanoma patients, the application of this signature could range from the initial diagnosis to monitoring and future studies could address these specific aspects achieving the transition from bench to bedside.

Our study also indicates an immunoregulatory role for miR-412-3p, miR-507 and miR-1203 that surely needs to be investigated further to fully elucidate their role.

Of note, the involvement in the tumorigenesis regulatory network of melanoma was already described for miR-507 [44]. MiR-412-3p, enriched in stem cells, was already described in a kidney epithelial tumor, as clear cell renal cell carcinoma (ccRCC) [45, 46], and colon cancer [47], as oncogenic microRNA favoring tumor growth and progression. Recently, miR-412-3p has been also reported as potential salivary EV biomarkers in oral squamous cell carcinoma [48].

MiR-362-3p has been previously described in human breast cancer [49], cervical adenocarcinoma [50] and renal cell carcinoma [51]. Furthermore, the prognostic value of miR-362-3p has been reported in squamous cell carcinoma, where lower miR-362-3p expression levels predicted unfavorable overall survival [52].

MiR-1203 has been identified as a potential serum biomarker for prostate cancer [53] and a predictor of prognosis for hepatocellular carcinoma [54].

Although our study presents limitations, due to the number of patients, our results support the idea that the 4 pEV-microRNA signature might have a powerful clinical relevance.

## Conclusions

To the best of our knowledge, this study provides for the first time the pEV-microRNA profiles from plasma samples of melanoma patients; the consistency of microRNA panel has been validated both in plasma samples from public data sets and EV-microRNA samples of an independent cohort. Our circulating microRNA signature offers a non-invasive tool for melanoma diagnosis, providing a novel strategy for improving standard care. Thus, our findings reveal a new tool for melanoma patients in the field of personalized medicine.

## Supplementary Information

**Additional file 1: Figure S1.** Snapshot of informative pEV-microRNAs of discovery cohort. **Figure S2.** Principal Component Analysis (PCA) of discovery cohort. **Figure S3.** Up-regulated pEV-microRNA dotplot of Gene Ontology (GO) enrichment analysis. **Figure S4.** Down-regulated pEV-microRNA dotplot of Gene Ontology (GO) enrichment analysis. **Figure S5**. Schematic representation of bioinformatics workflow for pEV-microRNA signature identification in metastatic melanoma cohorts. **Figure S6.** Heatmap of cell-type enrichment analysis.

**Additional file 2: Table S1.** A detailed list of differentially expressed pEV-microRNAs obtained from high-throughput PCR profiles in metastatic melanoma patients.

## Data Availability

All data generated and analysed during this study are included in this published article [and its Supplementary data files]. Public external data are available in the NCBI Gene Expression Omnibus under accession number GSE20994.

## Abbreviations

BP: Biological Processes
CPM: count per million
Ctrl: Controls
Ct: PCR cycle threshold
ddPCR: droplet digital PCR
EVs: Extracellular Vesicles
GO: Gene Ontology
pEVs: plasma Extracellular Vesicles
RT-qPCR: Reverse Transcription Quantitative Real-time PCR
ROC: Receiver Operating Characteristic
TEM: Transmission Electron Microscopy
WCL: whole cell lysate
