# Supplementary material for: A novel microRNA signature for the detection of melanoma by liquid biopsy
Source: J Transl Med. 2022 Oct 15;20:469. doi: 10.1186/s12967-022-03668-1 (PMC9571479; doi:10.1186/s12967-022-03668-1)

# **A novel microRNA signature for the detection of melanoma by liquid biopsy**

**ADDITIONAL MATERIAL**

***ADDITIONAL FIGURE LEGENDS***

**Additional Figure S1. Snapshot of informative pEV-microRNAs of discovery cohort.**

**(A)** Plot of informative pEV-microRNAs detected in all samples with a Ct ≤33 values. The red line denotes the chosen percentage of the number of samples (i.e. 10%, n=38). **(B)** Table showing for each percentage of the discovery cohort samples, the number of informative pEV-microRNAs that could be obtained; the green rectangle highlights for the percentage of samples selected (10%, n=38), the informative pEV-microRNAs (n=545).

**Additional Figure S2. Principal Component Analysis (PCA) of discovery cohort**.

The first two principal components (PCs) are plotted and colored according to sample category (Red: Ctrl, Cyan: Melanoma patients). PCA plots were performed using **(A)** raw pEV-microRNA data **(B)** and normalized pEV-microRNA data.

**Additional Figure S3. Up-regulated** **pEV-microRNA dotplot of Gene Ontology (GO) enrichment analysis.**

GO terms were grouped based on their semantic similarity and scores. The Gene Ontology- Biological Processes (GO-BP) are plotted in order of gene count, i.e. the number of microRNAs in the significant differentially expressed microRNAs list associated with the GO term. The size of the dots represents the gene ratio, i.e. the relative abundance of microRNAs in such a term and the color of the dots represents the log10 p-value. Enrichment for GO-BP terms among up-regulated pEV-microRNAs resulted from differentially expressed microRNAs between Patients and Ctrl.

**Additional Figure S4. Down-regulated pEV-microRNA dotplot of Gene Ontology (GO) enrichment analysis.**

GO terms were grouped based on their semantic similarity and scores. The Gene Ontology- Biological Processes (GO-BP) are plotted in order of gene count, i.e. the number of microRNAs in the significant differentially expressed microRNAs list associated with the GO term. The size of the dots represents the gene ratio, i.e. the relative abundance of microRNAs in such a term and the color of the dots represents the log10 p-value. Enrichment for GO-BP terms among down-regulated pEV-microRNA resulted from differentially expressed microRNAs between Patients and Ctrl.

**Additional Figure S5**. **Schematic representation of bioinformatics workflow for pEV-microRNA signature identification in metastatic melanoma cohorts.**

The model involves the identification of a diagnostic signature built on the differentially expressed pEV-microRNAs of the discovery cohort (on left), and further evaluated in the validation cohort (on right). The predictive model of 4 pEV-microRNAs (on middle) was able to distinguish melanoma patients respect to normal controls.

**Additional Figure S6. Heatmap of cell-type enrichment analysis.**

Heatmap showing the average expression values for 3 out 4 microRNAs (miR-412-3p; miR-507 and miR-362-3p) of the pEV-microRNA signature across cell types from Functional Annotation of Mammalian Genome (FANTOM5) project. (*) (For hsa-miR-1203 no expression data were available).

**Additional Figure S1**

**
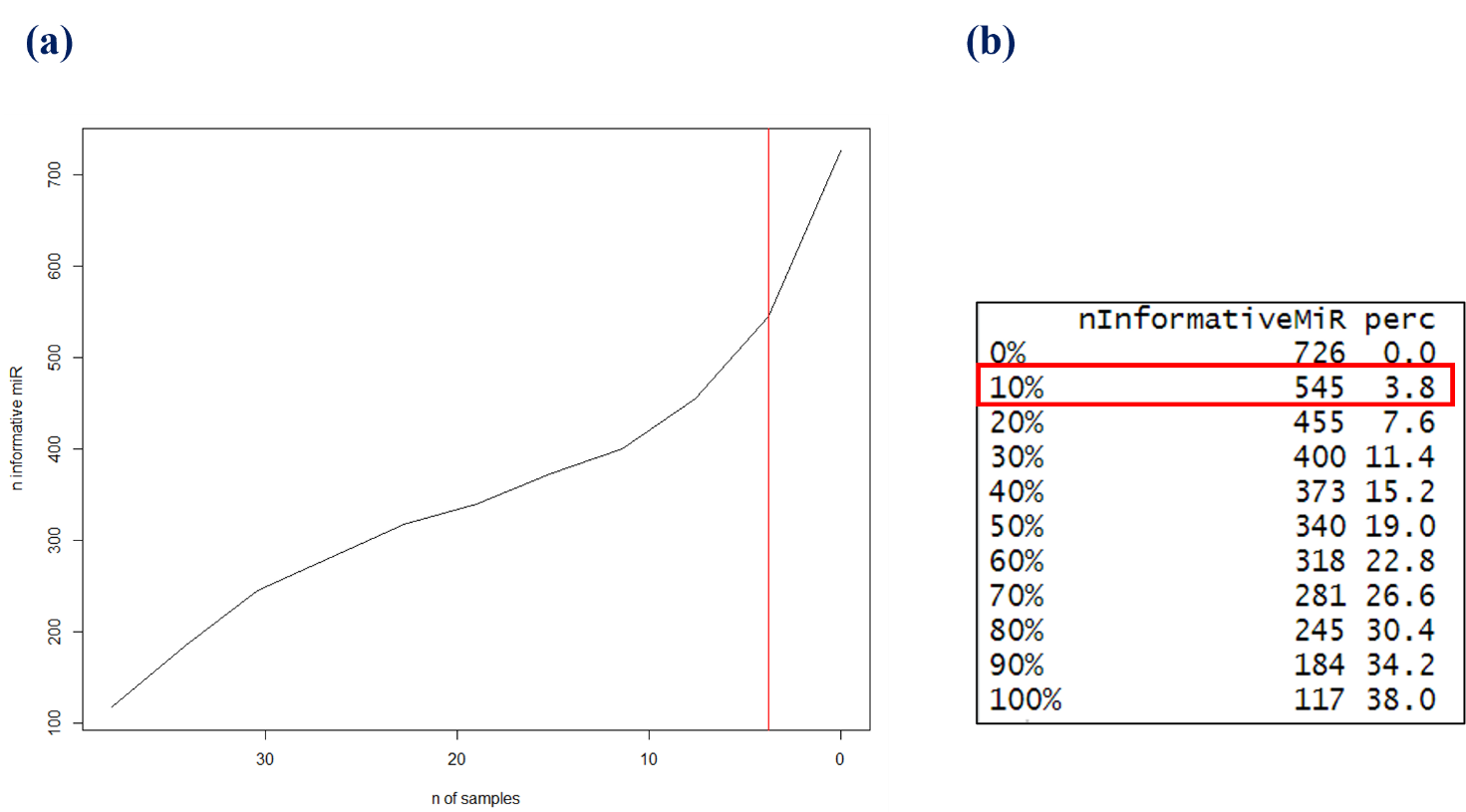
**

**Additional Figure S2**


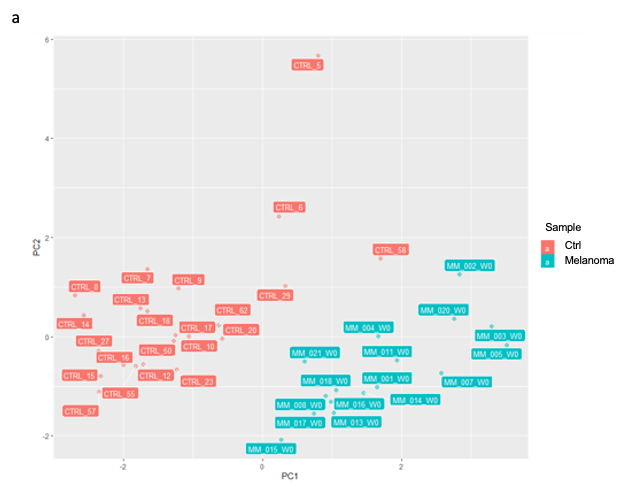


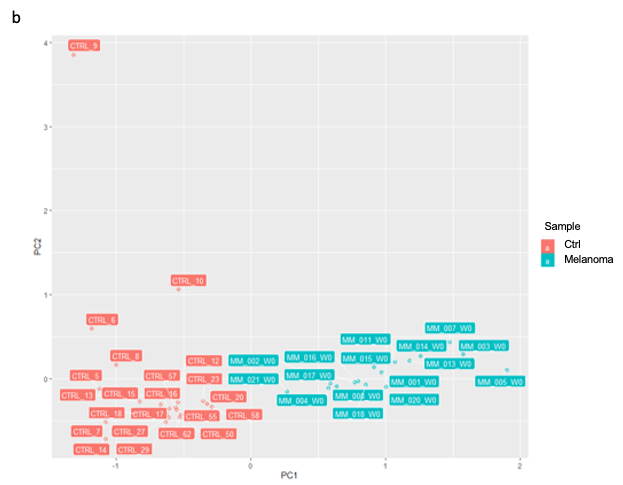


**Additional Figure S3**


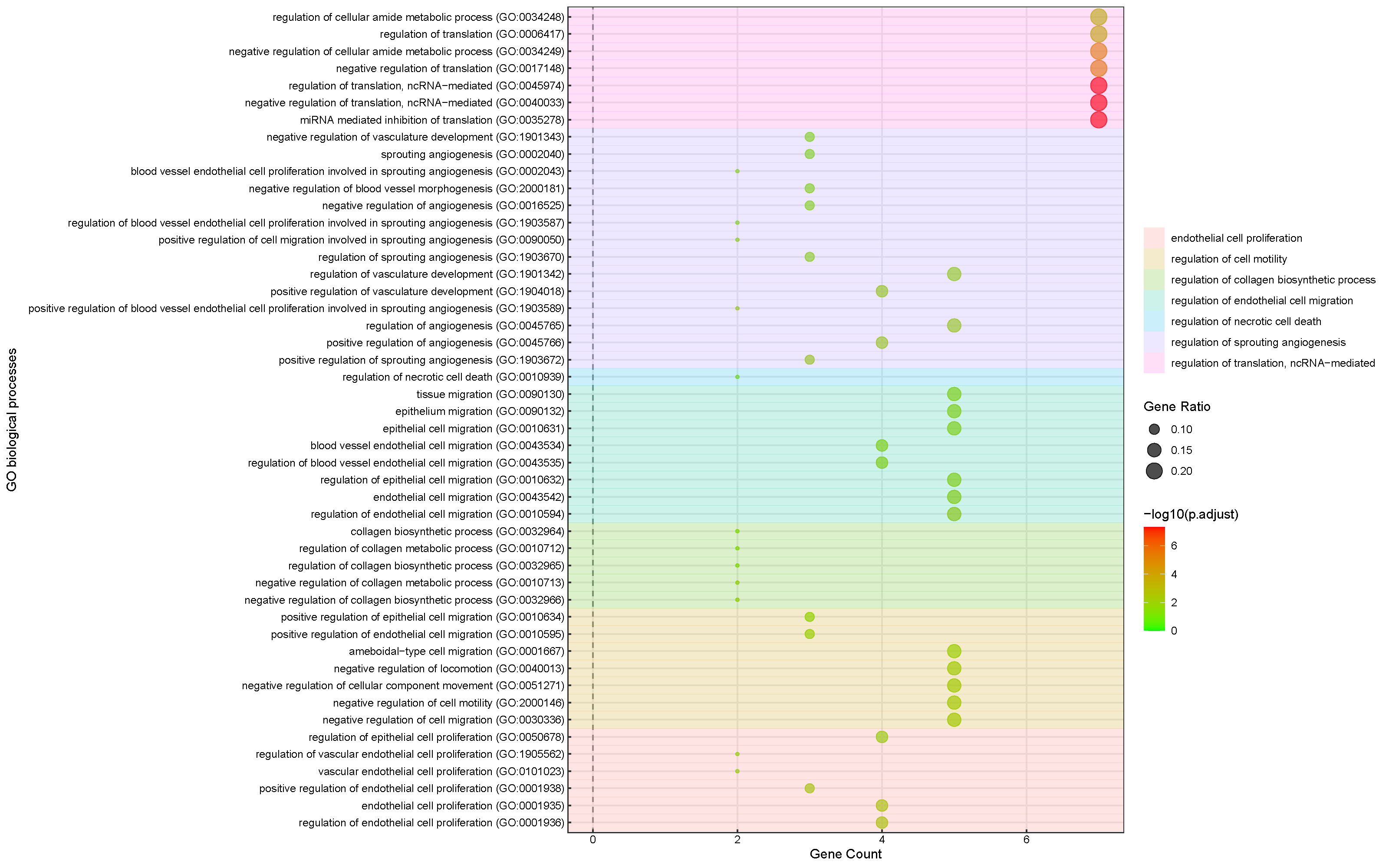


**Additional Figure S4**


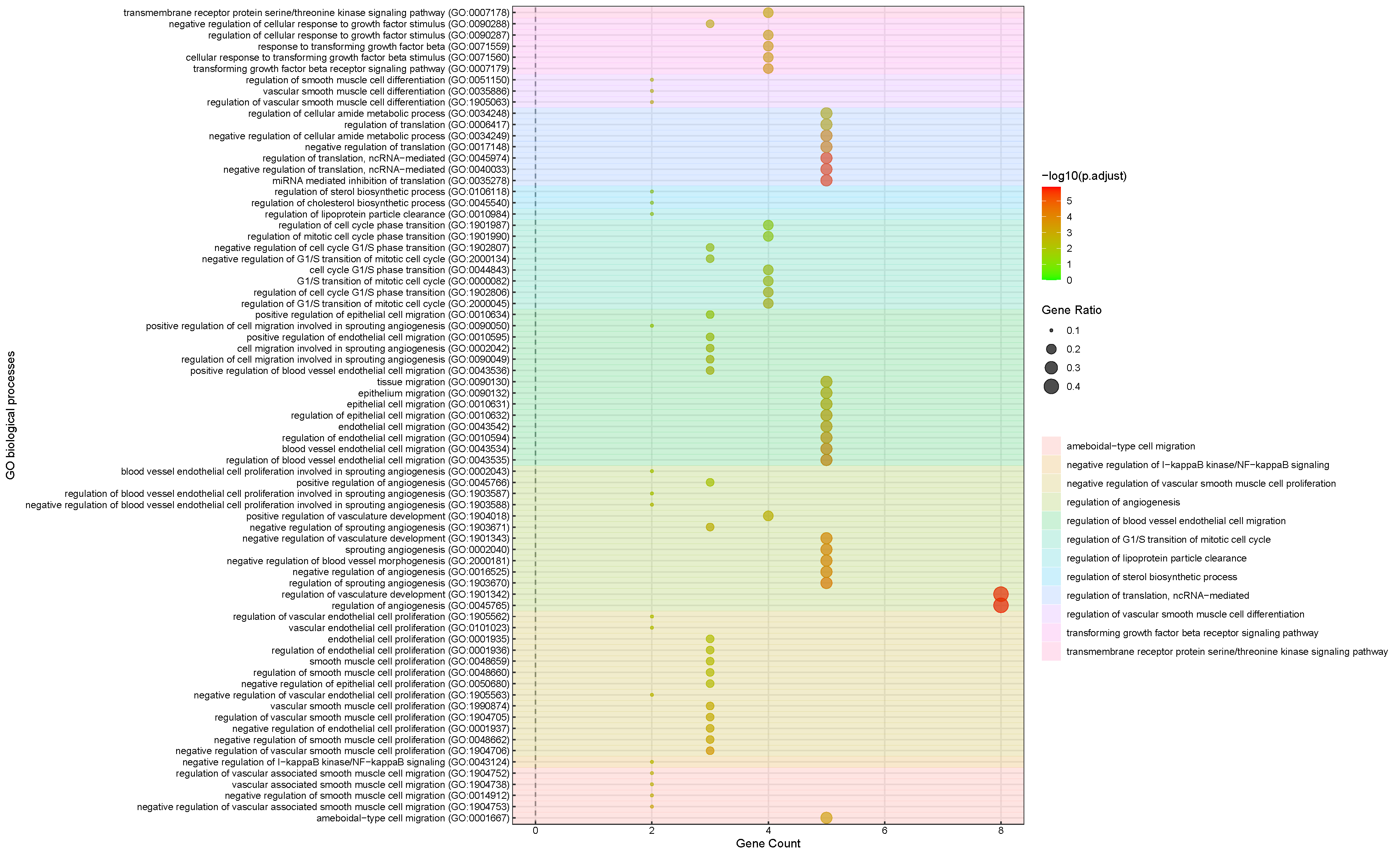


**Additional Figure S5**


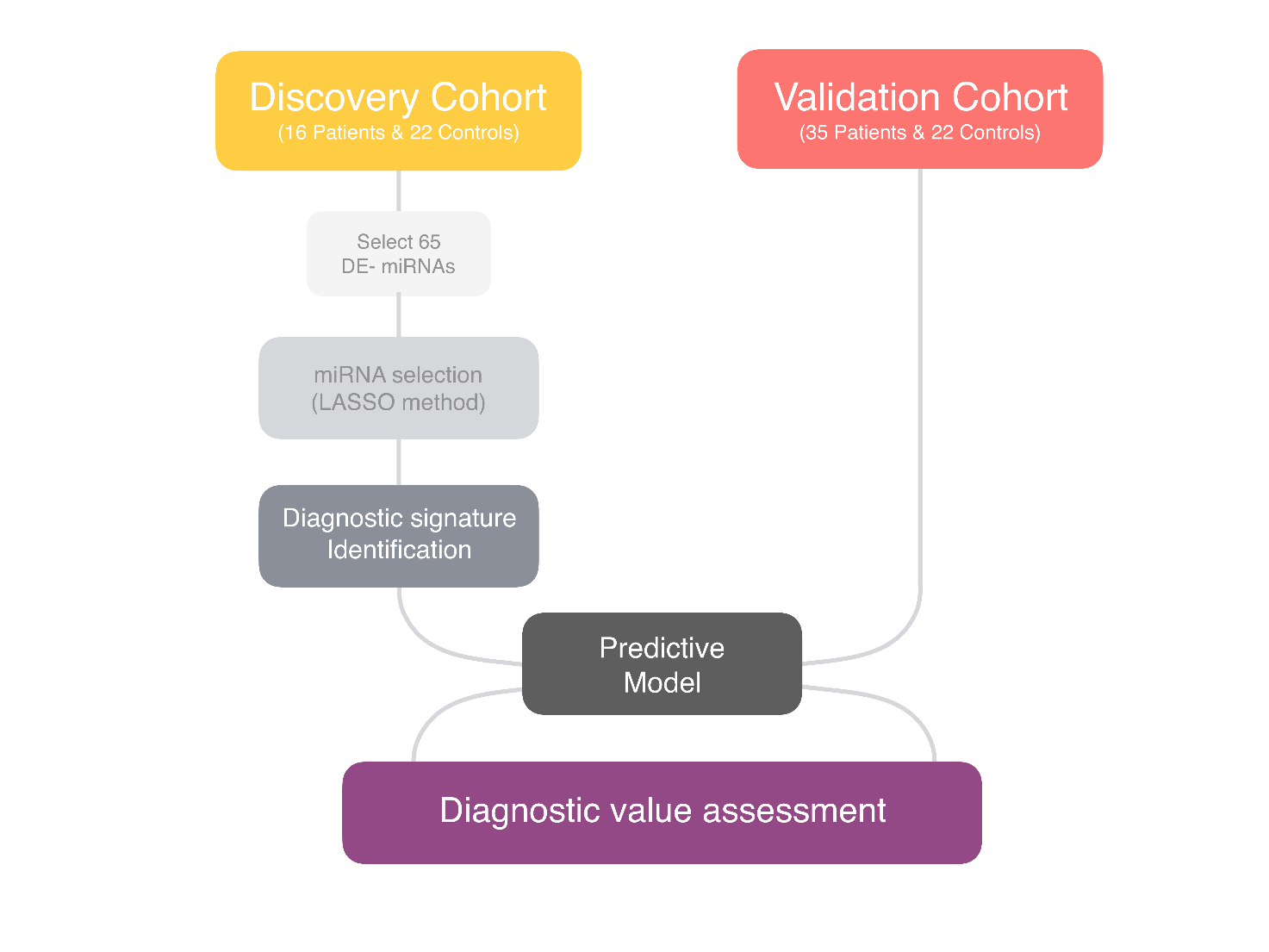
**Additional Figure S6**


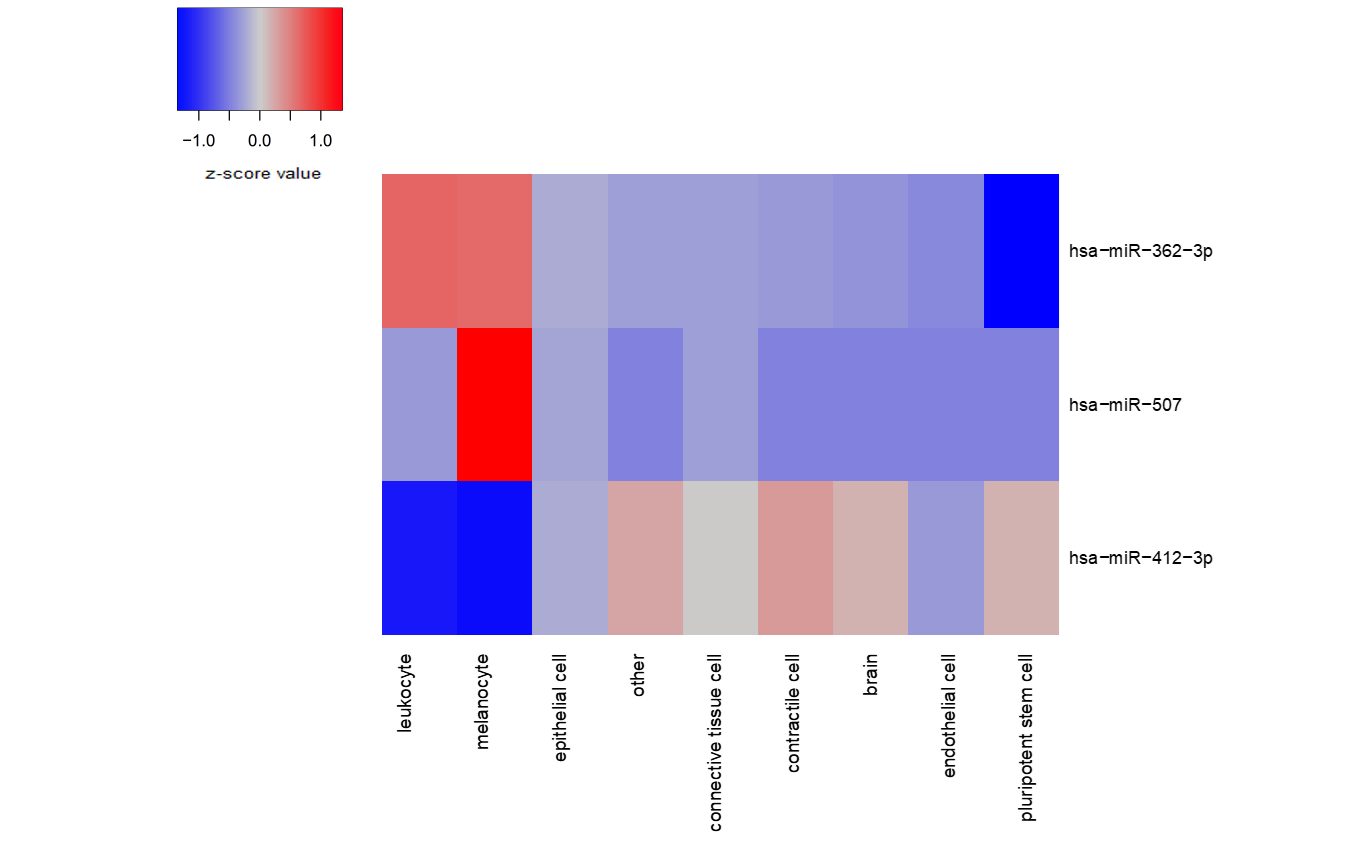

Supplement: Supplementary file 1 — Additional file 1: Figure S1. Snapshot of informative pEV-microRNAs of discovery cohort. Figure S2. Principal Component Analysis (PCA) of discovery cohort. Figure S3. Up-regulated pEV-microRNA dotplot of Gene Ontology (GO) enrichment analysis. Figure S4. Down-regulated pEV-microRNA dotplot of Gene Ontology (GO) enrichment analysis. Figure S5. Schematic representation of bioinformatics workflow for pEV-microRNA signature identification in metastatic melanoma cohorts. Figure S6. Heatmap of cell-type enrichment analysis. [file 12967_2022_3668_MOESM1_ESM.docx]
