# Supplementary material for: A novel microRNA signature for the detection of melanoma by liquid biopsy
Source: J Transl Med. 2022 Oct 15;20:469. doi: 10.1186/s12967-022-03668-1 (PMC9571479; doi:10.1186/s12967-022-03668-1)
Supplement: Supplementary file 2 — Additional file 2: Table S1. A detailed list of differentially expressed pEV-microRNAs obtained from high-throughput PCR profiles in metastatic melanoma patients. [file 12967_2022_3668_MOESM2_ESM.docx]

# **A novel microRNA signature for the detection of melanoma by liquid biopsy**

**ADDITIONAL MATERIAL**

***ADDITIONAL TABLE LEGENDS***

**Additional Table S1. A detailed list of differentially expressed pEV-microRNAs obtained from high-throughput PCR profiles in metastatic melanoma patients.**

The table reports the detailed list of 65 differentially expressed pEV-microRNAs in metastatic melanoma patients with respect to normal controls. Each microRNA was organized according to decreasing fold change (FC) values in the expression. For each microRNA p-values and their relative expression level (i.e. up-regulation; down-regulation) are also reported.

**Additional Table S1**

| ***microRNA*** | ***Fold Change (FC)*** | ***P-value*** | ***microRNA expression level*** |
| --- | --- | --- | --- |
| hsa-miR-1203-478310-mir | 100.539 | 1.7985e-10 | **Up-regulated** |
| hsa-miR-412-3p-478087-mir | 55.240 | 6.1510e-08 |  |
| hsa-miR-370-3p-478326-mir | 43.166 | 1.1678e-05 |  |
| hsa-miR-770-5p-479178-mir | 22.506 | 4.0134e-07 |  |
| hsa-miR-892a-479371-mir | 19.148 | 2.4460e-08 |  |
| hsa-miR-604-479085-mir | 19.074 | 5.1142e-07 |  |
| hsa-miR-450b-3p-478913-mir | 18.115 | 8.9928e-11 |  |
| hsa-miR-197-3p-477959-mir | 14.092 | 7.2955e-07 |  |
| hsa-miR-330-5p-478830-mir | 13.275 | 1.2853e-06 |  |
| hsa-miR-182-3p-478729-mir | 13.213 | 2.3706e-05 |  |
| hsa-miR-1228-5p-478644-mir | 11.739 | 0.0031 |  |
| hsa-miR-101-5p-478620-mir | 10.978 | 1.0791e-09 |  |
| hsa-miR-511-5p-478970-mir | 9.989 | 1.98381e-06 |  |
| hsa-miR-767-3p-479175-mir | 9.659 | 0.0016 |  |
| hsa-miR-507-478960-mir | 8.777 | 8.05819e-06 |  |
| hsa-miR-487a-3p-477826-mir | 8.428 | 1.4334e-07 |  |
| hsa-miR-554-479039-mir | 8.214 | 0.0224 |  |
| hsa-miR-616-3p-478177-mir | 7.922 | 0.0002 |  |
| hsa-miR-187-3p-477941-mir | 7.807 | 1.9838e-06 |  |
| hsa-miR-520g-3p-478991-mir | 7.782 | 0.0013 |  |
| hsa-miR-617-479100-mir | 7.471 | 0.0020 |  |
| hsa-miR-29a-5p-478002-mir | 7.018 | 0.0047 |  |
| hsa-miR-190b-477948-mir | 6.767 | 3.01376e-06 |  |
| hsa-miR-1267-478672-mir | 6.727 | 0.0010 |  |
| hsa-miR-636-478185-mir | 6.066 | 3.01376e-06 |  |
| hsa-miR-373-3p-478363-mir | 6.028 | 1.0791e-09 |  |
| hsa-miR-217-478773-mir | 5.953 | 4.04676e-09 |  |
| hsa-miR-9-5p-478214-mir | 4.978 | 0.0003 |  |
| hsa-miR-1183-477870-mir | 4.960 | 3.3247e-05 |  |
| hsa-miR-517c-3p-479487-mir | 4.958 | 0.0022 |  |
| hsa-miR-200c-3p-478351-mir | 4.945 | 8.2284e-08 |  |
| hsa-miR-27b-5p-478789-mir | 4.838 | 0.0034 |  |
| hsa-miR-1200-478631-mir | 4.779 | 0.0004 |  |
| hsa-miR-548b-3p-479018-mir | 4.771 | 0.0388 |  |
| hsa-miR-92a-1-5p-479205-mir | 4.766 | 1.2500e-08 |  |
| hsa-miR-628-3p-478181-mir | 4.736 | 2.6978e-09 |  |
| hsa-miR-643-479123-mir | 4.671 | 0.0010 |  |
| hsa-miR-1208-478637-mir | 4.476 | 0.0002 |  |
| hsa-miR-129-5p-477896-mir | 4.394 | 0.0160 |  |
| hsa-miR-558-479044-mir | 4.392 | 1.0791e-09 |  |
| hsa-miR-585-3p-479067-mir | 4.290 | 8.05819e-06 |  |
| hsa-miR-943-479219-mir | 4.289 | 0.01743 |  |
| hsa-miR-7-2-3p-478199-mir | 4.256 | 0.0042 |  |
| hsa-miR-432-5p-478101-mir | 4.066 | 0.0243 |  |
| hsa-miR-365a-3p-  hsa-miR-365b-3p-478065-mir | 0.2491 | 0.0012 | **Down-regulated** |
| hsa-miR-502-5p-478954-mir | 0.2419 | 7.4273e-05 |  |
| hsa-miR-15a-3p-477928-mir | 0.2360 | 1.6729e-05 |  |
| hsa-miR-183-5p-477937-mir | 0.23013 | 0.0003 |  |
| hsa-miR-425-5p-478094-mir | 0.2273 | 1.7985e-10 |  |
| hsa-miR-296-5p-477836-mir | 0.2140 | 3.5971e-10 |  |
| hsa-miR-185-5p-477939-mir | 0.21206 | 3.3543e-08 |  |
| hsa-miR-215-5p-478516-mir | 0.2078 | 8.1852e-07 |  |
| hsa-miR-144-3p-477913-mir | 0.1974 | 2.4460e-08 |  |
| hsa-miR-125b-5p-477885-mir | 0.1971 | 8.9928e-11 |  |
| hsa-miR-532-5p-478151-mir | 0.17205 | 1.7086e-09 |  |
| hsa-miR-144-5p-477914-mir | 0.16750 | 6.0251e-09 |  |
| hsa-let-7i-3p-477862-mir | 0.1565 | 6.2949e-10 |  |
| hsa-miR-660-5p-478192-mir | 0.1438 | 3.5971e-10 |  |
| hsa-miR-193b-3p-478314-mir | 0.1421 | 1.7535e-08 |  |
| hsa-miR-331-3p-478323-mir | 0.1387 | 0.0001 |  |
| hsa-miR-15b-3p-477929-mir | 0.1220 | 3.3543e-08 |  |
| hsa-let-7f-1-3p-477801-mir | 0.1174 | 4.0134e-07 |  |
| hsa-miR-30e-5p-479235-mir | 0.1146 | 4.5116e-06 |  |
| hsa-miR-30a-5p-479448-mir | 0.0948 | 0.0010 |  |
| hsa-miR-362-3p-478058-mir | 0.0472 | 8.9928e-11 |  |
